# Loss-to-follow-up and delay to treatment initiation in Pakistan’s national tuberculosis control programme

**DOI:** 10.1186/s12889-018-5222-2

**Published:** 2018-03-09

**Authors:** Syed Mustafa Ali, Farah Naureen, Arif Noor, Irum Fatima, Kerri Viney, Muhammad Ishaq, Naveed Anjum, Aamna Rashid, Ghulam Rasool Haider, Muhammad Aamir Khan, Javariya Aamir

**Affiliations:** 1 Mercy Corps, Rawal Chowk, Murree Road, Islamabad, Pakistan; 2 Australian National University, Canberra, Australia

**Keywords:** Pre-treatment loss to follow up, Lost to follow-up during diagnosis, Treatment delay, Tuberculosis, Pakistan’s TB control program

## Abstract

**Background:**

Researchers and policy-makers have identified loss to follow-up as a major programmatic problem. Therefore, the objective of this study is to quantify TB related pre-treatment loss to follow up and treatment delay in private sector health care facilities in Pakistan.

**Methods:**

This was a retrospective, descriptive cohort study using routinely collected programmatic data from TB referral, diagnosis and treatment registers. Data from 48 private healthcare facilities were collected using an online questionnaire prepared in ODK Collect, for the period October 2015 to March 2016. Data were analysed using SPSS. We calculated the: (1) number and proportion of patients who were lost to follow-up during the diagnostic period, (2) number and proportion of patients with pre-treatment loss to follow-up, and (3) the number of days between diagnosis and initiation of treatment.

**Results:**

One thousand five hundred ninety-six persons with presumptive TB were referred to the laboratory. Of these, 96% (*n* = 1538) submitted an on-the-spot sputum sample. Of the 1538 people, 1462 (95%) people subsequently visited the laboratory to submit the early morning (i.e. the second) sample. Hence, loss to follow-up during the diagnostic process was 8% overall (*n* = 134). Of the 1462 people who submitted both sputum samples, 243 (17%) were diagnosed with sputum smear-positive pulmonary TB and 231 were registered for anti-TB treatment, hence, loss in the pre-treatment phase was 4.9% (*n* = 12). 152 persons with TB (66%) initiated TB treatment either on the day of TB diagnosis or the next day. A further 79 persons with TB (34%) commenced TB treatment within a mean time of 7 days (range 2 to 64 days).

**Conclusion:**

Concentrated efforts should be made by the National TB Control Programme to retain TB patients and innovative methods such as text reminders and behavior change communication may need to be used and tested.

**Electronic supplementary material:**

The online version of this article (10.1186/s12889-018-5222-2) contains supplementary material, which is available to authorized users.

## Background

Pakistan is among 30 high tuberculosis (TB) burden countries globally, based on the absolute numbers of incident TB cases reported per annum [1]. Pakistan is also one of six countries globally that account for 60% of the world’s TB burden [1]. 510,000 incidence TB cases were estimated in 2015, resulting in an estimated TB incidence rate of 270 cases per 100,000 population [1]. In 2015, an additional 72,144 incident cases were notified by private healthcare facilities [1]. The TB mortality rate in Pakistan is also high at 23 cases per 100,000 population, however there has been a substantial decrease in TB mortality since the year 2000 [1]. In 2015, 323, 856 cases of TB were actually notified, resulting in an estimated 186,000 cases being “missed” by Pakistan’s National TB Control Programme. These cases comprise 36.5% of the estimated total TB burden and potentially contribute to ongoing TB transmission in the community.

In the Pakistan National TB Control Programme, pulmonary TB is diagnosed using sputum smear microscopy, whereby two sputum samples are collected and examined under a microscope for the presence of acid fast bacilli. This practice is consistent with international policies which recommend that at least one positive sputum smear is required to diagnose TB [2, 3]. Persons with extra pulmonary TB are diagnosed using clinical methods or by histopathology. After TB is diagnosed, the majority of patients start TB treatment shortly afterwards. However, a small proportion do not, and these patients are not captured in the routine recording and reporting system. By not recording these patients, programme effectiveness may be overestimated, as the total number of TB patients is not recorded [4]. Another group of patients are diagnosed but then experience delays in commencing TB treatment, which can lead to ongoing transmission of TB or other poor health outcomes [5–7]. According to the World Health Organization, an untreated TB patient can infect average 10–15 persons over the course of one year [8]. This figure emphasizes the importance of early diagnosis and subsequent enrolment onto TB treatment to render infectious patients non-infectious and to cure them of disease. Therefore, early detection, rapid diagnosis and the provision of high quality TB treatment are required to effectively control TB, as they help to interrupt ongoing transmission.

Researchers and policy-makers have identified pre-treatment loss to follow-up as a major programmatic problem. It contributes to the case detection gap, that is the number of people who have active TB and who are not detected by staff from national TB control programmes. Pre-treatment loss to follow up also very likely contributes to ongoing transmission of TB, hampering efforts by national TB control programmes to effectively control the disease.

According to the diagnosis and care pathway developed by MacPherson et al. (2014), loss to follow-up (LTFU) can occur at multiple stages of the care pathway, including LTFU during the diagnostic period (i.e. in the period of time during which the patient is being diagnosed, after referral from a clinic or health facility), pre-treatment loss to follow-up (i.e. after diagnosis and before TB treatment commences) and on-treatment lost to follow-up (i.e. during TB treatment). The proportion of TB patients who are lost to follow up prior to starting TB treatment ranges between 4 and 38%, and is higher in sub-Saharan Africa (18%) than in Asia (13%) [9].

For the patients who do start TB treatment, another significant problem is the delay in starting this treatment, after diagnosis. Ideally, all TB patients who are diagnosed should start TB treatment as soon as possible after diagnosis, however, for various reasons, many do not. Additional morbidity, ongoing transmission and death can result from this delay [5–7].

There have been limited studies on this issue in the Pakistani context, particularly in the private sector. Therefore, we conducted a study on TB related pre-treatment loss to follow up and treatment delay in private sector health care facilities in Pakistan. Our main objectives were to quantify: (i) the number and proportion of TB patients who were lost to follow-up, during the diagnostic period, (ii) the number and proportion of TB patients who were lost to follow-up after diagnosis and pre-treatment; and (iii) the duration of time between TB diagnosis and treatment initiation, to determine the number and proportion of TB patients who experienced treatment delay.

## Methods

### Design

This was a retrospective, descriptive cohort study using routinely collected programmatic data from TB referral, diagnosis and treatment registers.

### Setting and participants

The setting was 48 private healthcare facilities in Pakistan (24 facilities in four districts in Punjab Province (Hafizabad, Jhang, Mandi Bahudin and Narowal districts) and 24 facilities in four districts in Sindh Province (Badin, Ghotki, Khairpur and Shikarpur districts). The participating health centres were part of a Public Private Mix (PPM) project, managed by Pakistan’s National TB Control Programme. As part of this project, Mercy Corps, an international Non-Governmental Organization, provided training, medicines and other incentives for patients seeking TB care in private health care facilities. A total of 2000 health care facilities belong to this project spread across 75 districts of Pakistan. The selection of the 48 participating healthcare facilities was based on programmatic indicators, i.e., the number of referrals for persons with presumptive TB and the number of registered TB patients (including those who were sputum smear positive). Healthcare facilities that performed better on these indicators were selected to participate in this study. At the participating healthcare facilities, we collected the records of all people with presumptive TB, from the referral, diagnosis and treatment registers, for the period October 2015 to March 2016.

### The care pathway for persons with tuberculosis

In Pakistan, people who present to the outpatients department at a healthcare facility with signs and symptoms of TB are referred to the laboratory for sputum smear microscopy, where they are asked to produce (two sputum samples). At the outpatients department the person’s details are recorded in a referral register and a copy of this referral is handed to the patient to take to the laboratory. One of the sputum samples is collected on-the-spot, i.e., when a person with presumptive TB visits the laboratory and the second sputum sample is collected early the next morning (in order to yield a higher concentration of bacilli) [10]. The person with presumptive TB then drops off the sputum samples to the laboratory, and the details of this person are recorded in the laboratory register. Subsequently, sputum smear microscopy is performed and the results, whether positive or negative, are recorded in a diagnostic register. A patient with sputum smear-positive TB is referred back to the clinic with the diagnostic report, and standardized TB treatment is initiated according to the National TB Control Programme guidelines [11]. Persons with sputum smear negative TB are referred back to the clinic for further clinical investigations, according to the National TB Control Programme guidelines [11]. The patients are then recorded in the treatment register. Throughout this process, people with presumptive TB and also people who are subsequently diagnosed with TB can be lost to follow up and the staff from the Pakistan National TB Control Programme are not sufficiently resourced to follow up all of these people, even when they are sputum smear positive.

**Fig. 1:**
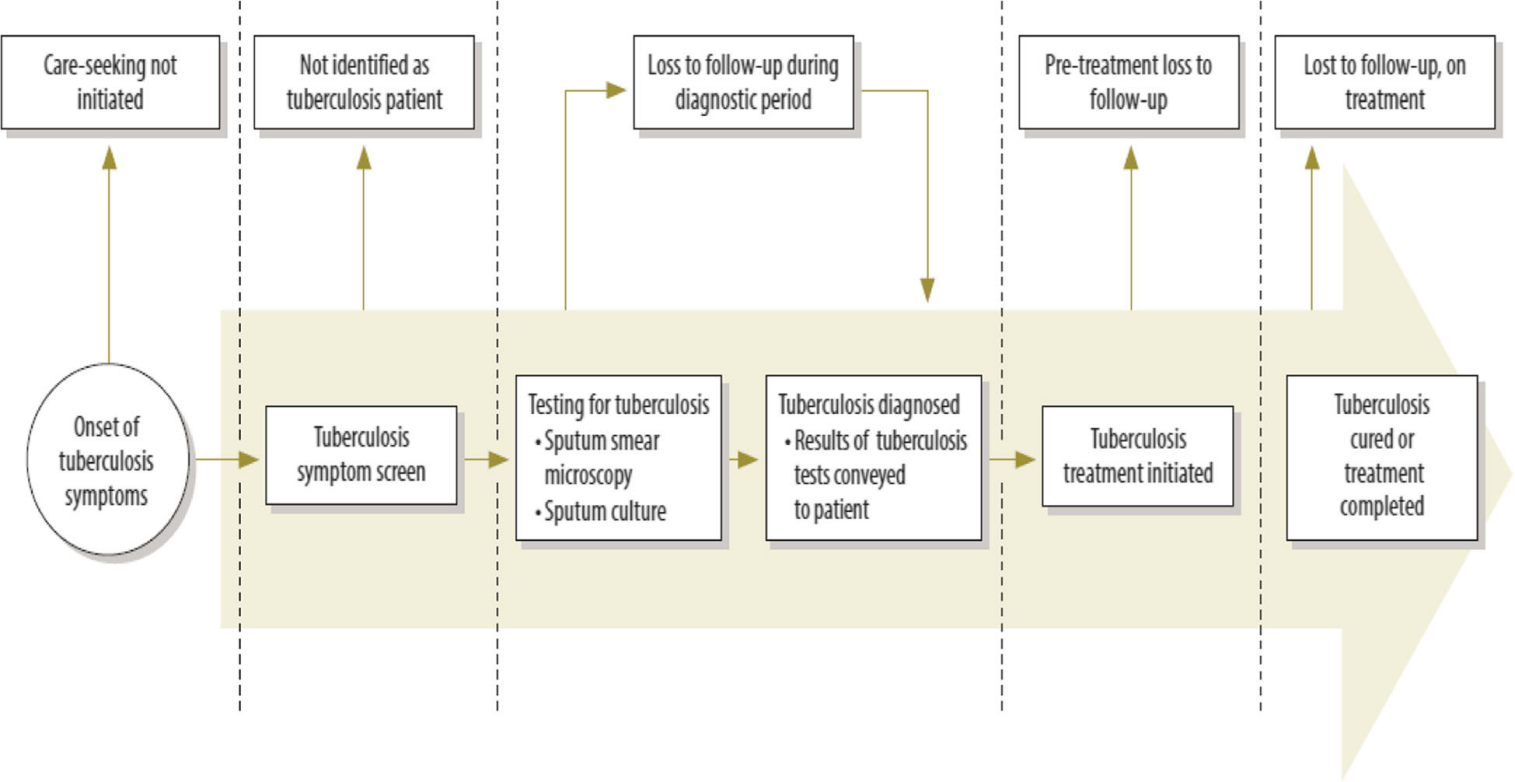
The Care Pathway for Tuberculosis Prevention and Care (adopted from MacPherson et al. 2014)

The TB care pathway, developed by MacPherson et al. (2014), outlines the different stages of TB care and different types of loss to follow-up experienced by patients; our study followed the definitions in this pathway (Fig. 1) [9]. In our study we aimed to quantify two types of loss to follow up: 1) loss to follow up during the diagnostic period, and 2) pre-treatment loss to follow up. We defined loss to follow up during the diagnostic period as the proportion of persons with presumptive TB who submitted at least one sputum to the laboratory, who were “lost” between the clinic and the laboratory. Therefore, the numerator was the number of persons with presumptive TB who had at least one sputum specimen recorded in the laboratory register and the denominator was the number of persons with presumptive TB who were referred to the laboratory for diagnostic testing. We defined pre-treatment loss to follow as the proportion of patients who were diagnosed but who did not commence TB treatment. Therefore, the numerator was the number of TB patients who commenced TB treatment during the study period and the denominator was the number of TB patients who were diagnosed with TB.

Additionally, we assessed treatment delay by enumerating the number of days between the date of diagnosis and the date of commencement of TB treatment. We included sputum smear positive TB patients only in this sample. As, there is no standard definition of treatment delay [12], for this study, delay of more than 4 weeks or 30 days was considered as treatment delay as used in most of the studies.

### Data collection and analysis

Data collected from the referral, diagnostic and treatment registers were cleaned, and inconsistencies (i.e. incorrect or incomplete values) were detected and if possible, rectified. Data were then entered into an online questionnaire prepared in ODK Collect and were analysed using SPSS (IBM Corp. Released 2011 SPSS Statistics for Windows, Version 20.0 Amonk, NY: IBM Corp.). We calculated the: (1) number and proportion of patients who were lost to follow-up during the diagnostic period, (2) number and proportion of patients with pre-treatment loss to follow-up, and (3) the number of days between diagnosis and initiation of treatment.

## Results

### Loss to follow up during diagnosis

**Fig. 2:**
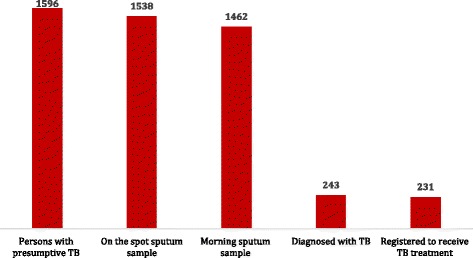
Tuberculosis Cascade of Care

During the study period, 1596 persons with presumptive TB were identified from the referral register and were referred to the laboratory (Table 1). Of these, 96% (*n* = 1538) submitted an on-the-spot sputum sample. The majority of those who did not submit the on-the-spot specimen were male (74%, *n* = 43). For the submission of the early morning sputum sample, of the 1538 people who submitted an on-the-spot sputum sample, 1462 (95%) people subsequently visited the laboratory to submit the early morning (i.e. the second) sample. Therefore, during the diagnostic process, of the 1596 persons with presumptive TB, a total of 134 people did not submit either one or both sputum samples, hence loss to follow-up during the diagnostic process was 8% overall (*n* = 134) (Fig. 2). Two thirds (66%, *n* = 89) of these people were male.

**Table 1:** Characteristics of Study Participants

Characteristics	Number (percentage)
Gender	
Male	948 (59)
Female	647 (41)
Not recorded	Nil
District	
Hafizabad	300 (19)
Jhang	160 (10)
Mandi Bahaudin	157 (10)
Narowal	178 (11)
Badin	153 (10)
Ghotki	150 (9)
Kahirpur	196 (12)
Shikarpur	301 (19)

### Pre-treatment loss to follow-up

Of the 1462 people who submitted both sputum samples, 243 (17%) were diagnosed with sputum smear-positive pulmonary TB. Of these, 231 (95%) were registered for anti-TB treatment. Therefore, of the 1596 persons with presumptive TB, the proportion who were lost in the pre-treatment phase was 4.9% (12/243) (Fig. 2). A greater proportion of females (*n* = 7; 58%) were lost during this phase.

### Treatment delay

Of the 231 patients with sputum smear-positive pulmonary TB, 152 (66%) initiated TB treatment either on the day of TB diagnosis or the next day. A further 79 (34%) commenced TB treatment within a mean time of 7 days (range 2 to 64 days). Among these 79 patients, more patients were from the Punjab (62%, *n* = 49) than Sindh (38%, *n* = 30). Such cases were evenly distributed between males (*n* = 40; 51%) and females (*n* = 39; 49%). Moreover, 22% (18 out of 79) of these patients started treatment after 7 days (range 9 to 64 days). However, there were only 5 patients who had delayed treatment initiation as they started anti-TB treatment after 4 week cut off point.

## Discussion

The findings of this study indicate that loss to follow-up is slightly higher in the diagnosis period than the pre-treatment period. Once diagnosed with TB, two thirds of patients initiate TB treatment on time. However in total, 79 sputum smear-positive pulmonary cases had a delayed start to treatment, with a mean delay of 7 days.

We observed 8% loss to follow-up during the diagnostic period and nearly 5%in the pre-treatment period. However, another study in Pakistan reported 12.9% loss to follow up, of which nearly 5.2% was pre-treatment [13]. In similar studies in Pakistan, Fatima et al. (2011), Rao et al. (2011) and Rao et al. (2009) reported 16,145, 7467 and 869 presumptive cases respectively. Of these, the respective LTFU rates were 6%, 15% and 28% [14–16]. Moreover, in other Asian countries, such as India [17], Vietnam [18] and Tajikistan [19] LTFU rates have been recorded as 4%, 8% and 18%, respectively. Loss to follow-up rate in the private sector who report to Pakistan’s National TB Control Programme is slightly lower, probably because of the oversight approach employed. At field or district level, focal persons from public and private sides hold regular quarterly meetings to keep track of the performance. To support this, a district-level TB register is maintained by field staff and it is ensured that no patient is dropped during the TB care pathway.

Patient counselling is seen as important intervention to reduce LTFU, however, it is unclear whether counselling has influenced our results. In one study in Pakistan, patient counselling did not impact on the rate of LTFU [13]. Other studies have shown that behavioral counselling integrated with TB care generated better adherence to treatment protocols overall [20, 21] and patients perceive counselling as a valuable inclusion in TB control programmes, especially when counselling is combined with financial support [21].

Addressing communication gaps among TB clinics, laboratories and patients may be one solution to these types of programmatic problems [22]. The emerging mHealth technologies may possibly bridge the communication gap between health care providers and patients and could reduce the rate of LTFU [23, 24], for example, text message-based interventions [25, 26]. However, to allow improved mobile phone communication to happen, contact details need to be complete in TB registers, which is often not the case [27].

Another possible reason for LTFU is that people with presumptive TB need to wait for results. In a discussion paper where 100 hypothetical patients were diagnosed with TB, Davis et al. (2012) found that same-day diagnosis increased the chance of treatment initiation when compared to sputum collection over one or two days, with reporting of results afterwards [28]. Moreover, a meta-analysis of eight studies revealed that the sensitivity of same-day sputum microscopy versus standard smear microscopy was similar [29]. Furthermore, advancements in mobile phone based microscopy may also offer great opportunities to facilitate timely interpretation and communication of sputum smear results [30, 31].

The use of molecular diagnostic tests and same day reporting and TB treatment initiation may also reduce LTFU. In the districts in which we carried out our study, microscopy remains the mainstay of diagnosis. Considering the microscopy-specific diagnostic algorithm, the introduction and roll out of molecular tests such as the Xpert MTB/RIF test is promising [32]. The Xpert MTB/RIF test provides quick and accurate diagnosis that can potentially reduce delays and dropouts during the diagnostic process [33]. For example, the median time to treatment initiation for patient diagnosed using Xpert MTB/RIF in South Africa was 0 days, whereas, for other forms of diagnosis using radiology and culture, the median times to treatment initiation were 14 days and 144 days, respectively [34].

We studied loss to follow up during the diagnosis period and then before treatment initiation. During the diagnostic period, more males were lost compared to females. In another study, carried out in one district of Pakistan, 3.4% more males were lost, than females (16.5% vs 13.1%) [35]. There are well recognized differences in the gender ratio of TB patients, with males outnumbering females by 12% [1]. These differences may be due to health seeking behavior and socio-economic factors. For example, Malawian females are more conscious about their health and would visit a healthcare facility earlier than men [36]. However, men may also be given priority over women when accessing healthcare facilities [37, 38]. Additionally, lower TB case notification rates among women may be due to stigma, restricted access to financial resources and traditional beliefs [38–40]. Higher case notification rates among men may also be due to a higher prevalence of risk factors for TB among men, such as cigarette smoking, exposure to silica dust, etc. [41].

The strength of this study is a rigorous approach of case ascertainment across three TB registers, i.e., the referral register, the diagnostic register and the TB treatment register. However, missing data was a limitation of the study. We also focused on pulmonary TB and sputum smear positive TB only and did not include information on referral, diagnosis and treatment for clinically diagnosed pulmonary patients and extra-pulmonary TB patients. Additionally, people who were lost to follow up were not tracked in TB registers of the neighboring districts to ascertain if the patients accessed care elsewhere, and might have initiated their TB treatment elsewhere. In addition, including “well performing” private care providers in a sample, we may have biased our findings, which could be different if a representative sample of private care providers was taken. As, our study used a non-representative sample, so it may be difficult to generalize our findings to the whole of Pakistan.

## Conclusion

We observed an 8% loss to follow up during the diagnostic period and a further 4.9% between diagnosis and commencement of TB treatment. Concentrated efforts should be made by the National TB Control Programme to retain these patients and innovative methods such as text reminders may need to be used and tested. Actions to improve communication between care stakeholders (clinicians, laboratory technicians and patients) should be prioritized and additional resources should be used to initiate anti-TB treatment without any delay. In addition, influencing positive behavior change in patients through care-centric awareness campaign including information on TB symptoms, its prevention and treatment can be considered an effective strategy in reducing diagnostic and treatment delays.

## Additional file

Additional file 1: De-identified data taken from patient registers. (XLSX 413 kb)
